# Benefits of Zero-Phase or Linear Phase Filters to Design Multiscale Entropy: Theory and Application

**DOI:** 10.3390/e26040332

**Published:** 2024-04-14

**Authors:** Eric Grivel, Bastien Berthelot, Gaetan Colin, Pierrick Legrand, Vincent Ibanez

**Affiliations:** 1 IMS Laboratory, Bordeaux INP, Bordeaux University, UMR CNRS 5218, 33400 Talence, France; 2 Thales AVS France, Campus Merignac, 75-77 Av. Marcel Dassault, 33700 Mérignac, France; bastien.berthelot@fr.thalesgroup.com (B.B.); vincent.ibanez@fr.thalesgroup.com (V.I.); 3 ENSEIRB-MATMECA, Bordeaux INP, 33400 Talence, France; 4 IMB Laboratory, Bordeaux University, UMR CNRS 5251, ASTRAL Team, INRIA, 33400 Talence, France; pierrick.legrand@u-bordeaux.fr

**Keywords:** MSE, linear-phase filter, coarse-grained, entropy rate

## Abstract

In various applications, multiscale entropy (MSE) is often used as a feature to characterize the complexity of the signals in order to classify them. It consists of estimating the sample entropies (SEs) of the signal under study and its coarse-grained (CG) versions, where the CG process amounts to (1) filtering the signal with an average filter whose order is the scale and (2) decimating the filter output by a factor equal to the scale. In this paper, we propose to derive a new variant of the MSE. Its novelty stands in the way to get the sequences at different scales by avoiding distortions during the decimation step. To this end, a linear-phase or null-phase low-pass filter whose cutoff frequency is well suited to the scale is used. Interpretations on how the MSE behaves and illustrations with a sum of sinusoids, as well as white and pink noises, are given. Then, an application to detect attentional tunneling is presented. It shows the benefit of the new approach in terms of *p* value when one aims at differentiating the set of MSEs obtained in the attentional tunneling state from the set of MSEs obtained in the nominal state. It should be noted that CG versions can be replaced not only for the MSE but also for other variants.

## 1. Introduction

In statistical signal processing, a standard processing chain consists of extracting some markers from the data initially collected with sensors. These markers make it possible to characterize the signal samples, which sometimes describe a physical phenomenon. For instance, in biomedical applications, the power in some frequency bands can be representative of the activity of the sympathetic or parasympathetic nervous system or the activity of the brain. As an alternative, the practitioner can search for an a priori model to represent the data using a small set of parameters. Thus, according to the Wold decomposition, moving average (MA), autoregressive (AR), ARMA, fractionally integrated (FI), and also ARFIMA processes can be considered. They can be seen as the filtering of a white noise. The first ones allow the short-memory processes to be modeled, while the last ones are relevant for long-memory processes. Discrete fractional Gaussian noise (dfGn) and discrete fractional Brownian motion (dfBm) can be also of interest. Sums of complex exponentials can be also well suited to some signals whose spectrum is discrete. Whatever the model, the model parameters are related to the power spectral density (PSD) and consequently to the correlation function. Other markers are used. For instance, they are related to the regularity and the self-similarity property of the signal under study. A time-continuous process y(t) is said to be self-similar with the Hurst parameter *H* if and only if:

(1) $$x\left(Nt\right)\overset{d}{=}N^{H}x\left(t\right)$$

where x(Nt) is the timescaled signal by a scale factor *N*, and $\overset{d}{=}$ means an equivalency in terms of the distribution. In that case, the Hurst exponent can be estimated by using different approaches such as the fluctuation analysis, the detrended fluctuation analysis, or some variants. See for instance [1,2,3,4,5].

In this paper, we propose to focus our attention on another marker, namely the sample entropy (SE or *Sampen*). In the Appendix A.1, we recall some successive steps that led to the SE. For a vector ${\underline{x}}_{1}$ storing the samples ${\left\{x_{n}\right\}}_{n=1,\ldots N}$ of the signal *x*, the SE can be expressed as

(2) $$\begin{array}{c} SE\left({\underline{x}}_{1},\tau ,m,r\right)=-\log\frac{P_{r}^{m+1}\left(\left[\begin{array}{ccc} x_{1} & x_{1+\tau }\cdots & x_{1+\left\lfloor \frac{N-1}{\tau }\right\rfloor \;\tau } \end{array}\right]\right)}{P_{r}^{m}\left(\left[\begin{array}{ccc} x_{1} & x_{1+\tau }\cdots & x_{1+\left\lfloor \frac{N-1}{\tau }\right\rfloor \;\tau } \end{array}\right]\right)} \end{array}$$

where $\left\lfloor .\right\rfloor$ is the floor function. In addition, $P_{r}^{m}\left(\left[\begin{array}{ccc} x_{1} & x_{1+\tau }\cdots & x_{1+\left\lfloor \frac{N-1}{\tau }\right\rfloor \;\tau } \end{array}\right]\right)$ is the estimated probability that the distance between two different vectors storing *m* consecutive values of the sample set is smaller than the tolerance level *r*.

Among the results that were established, the SE is known to be less sensitive to the noise than the approximate entropy (ApEn) [6]. In addition, the SEs of white noise and 1/f noise were studied for different scales τ. An analytical expression of the SE for unit variance zero-mean white noise was obtained [7,8]:

(3) $$\begin{array}{cc} \; & SE\left({\underline{x}}_{1},\tau ,m,r\right)=-\log\;\left(\;\int_{-\infty }^{+\infty }\;\;\sqrt{\frac{\tau }{8\pi }}\;\left[erf\;\left(\;\frac{x+r}{\sqrt{\frac{2}{\tau }}}\;\right)\;-erf\;\left(\;\frac{x-r}{\sqrt{\frac{2}{\tau }}}\;\right)\;\right]\;e^{-\frac{1}{2}x^{2}}dx\;\right) \end{array}$$

where $erf\left(x\right)=\frac{2}{\sqrt{\pi }}\int_{0}^{x}exp\left(-t^{2}\right)dt$ refers to the error function. Given (3), the SE of white noise monotonically decreases with τ, whereas the SE of CG 1/f noise is almost constant. In addition, the SE is sensitive to short-duration signals. Indeed, Richman mentioned that more than $10^{m}$ samples are required to get a “good” estimation of $SE({\underline{x}}_{1},\tau ,m,r)$.

However, distinguishing the interbeat interval time series of different diseased and healthy states is not necessarily an easy task if only a single scale of the signal is considered. In addition, there is no clear relationship between the entropy-based regularity and the “complexity” (it should be noted that different “complexity” measures of random sequences were proposed in the literature: the linear complexity, the maximum-order complexity, the nonlinear complexity [9], the 2-adic complexity [10], the Lempel–Ziv (LZ) complexity [11,12] in which the signal is converted to a binary sequence by comparing the signal with threshold(s) determined by CG methods (the mean, the median, the midpoint, or the k means), some variants of the LZ complexity such as its extension to the multiscale [13], the permutation LZ complexity [14] and its extensions to the multiscale [15,16], the dispersion LZ complexity and its extension to the multiscale analysis [17], the eigen complexity [18,19], the statistical complexity based on the Rényi entropy [20], and the T complexity. Moreover, some studies were conducted to define the relationship between complexity measures and Shannon entropy. See for instance [18]). Indeed, when the above quantity was directly used with physiologic signals and more particularly heartbeat interval series, larger values were obtained from pathological subjects than healthier ones. However, the contrary was rather expected by the experts, as the complexity is related to the ability of living systems to adapt themselves to the situation. Therefore, different scales of the signal were considered [7]. This led to the multiscale entropy (MSE) [7]. It has been used in a wide range of applications and more particularly in biomedical applications, traffic time series, real vibration data, diagnosing rotating machinery faults, and financial markets. Different families of MSE have been proposed for the last decades. The reader can refer to the Appendix A.2 to get more details.

The standard version of the MSE consists of estimating the SE of the process at various “scales”. These sequences are obtained from a coarse-graining (CG) process. Initially, a CG process is known to be a way to obtain robust information of a dynamical system by mapping original signals into symbol sequences. The mapping can be based on the rounding, min, max, midpoint, and averaging operators. When dealing with the latter and from a signal processing point of view, the signal is filtered by an averaging finite impulse response (FIR) causal filter whose order defines the scale. The frequency response of the filter is low-pass, and so all the more as the scale is high. Then, the filter output is decimated by a factor equal to the scale (in [21], the time-shift MSE consists of computing the SE of the original data but also the mean of the SEs of the τ sequences deduced from the data decimated by a factor τ, with τ chosen up to a maximum value. Although the author motivates the design of this variant of the MSE by drawing inspiration from Higuchi’s fractal dimension, it corresponds to the composite MSE (cMSE) [22] without the averaging step (See (A3)). Once again, the consequence of the decimation is not taken into account). Using this CG process could be a priori relevant for multifractal analysis or when dealing with processes such as dfGn whose normalized covariance function remains the same after CG. However, the signals processed in practice are unlikely to have these properties. In [23], Zhang combines CG and SE in order to introduce the concept of the complexity measure, which is between regular and completely disordered data. His aim was to show that the sum of the sample entropies evaluated on the signal and their CG versions is maximized when the signal that is studied is a 1/f noise signal. As mentioned in [24,25,26], the decimation may be problematic if the design of the filter is not done properly. Indeed, the Shannon theorem should be satisfied for each scale. Although aliasing is a priori avoided in [25,26,27] by using a Butterworth filter with a well-suited cutoff frequency, the phase distortions induced by the filter in the pass band can be a source of problems. In this paper, we propose to consider two types of solutions: the first one consists of designing a linear-phase FIR filter. The window method or the Remez algorithm can be used, and we will see which method is the most relevant. The second one is based on an infinite-impulse filter (IIR)-based structure, which leads to a null-phase equivalent filter. Three studies are then conducted: one based on a sum of sinusoids, a second one on white and pink noises, and the last one on real data in order to detect the tunneling of aircraft pilots, which corresponds to a cognitive state during which the pilots are unable to detect visual alarms because they are focused on the task to be done. We will see that our approach can be more relevant than the standard CG-based MSE.

The remainder of this paper is organized as follows. In Section 2, the MSE based on the linear-phase or null-phase filter is first introduced. In Section 3, illustrations are given with white noise and 1/f noise. More particularly, we present how the measure evolves with the scale based on a certain number of trials. We also show the relevance of our approach with a sum of sinusoids. As the SE is defined from the ratio of two probabilities, we propose to better understand the link between both probabilities. Finally, the application on real data is proposed. It should be noted that this paper is an extension of a conference paper [28].

## 2. MSE Based on a Linear-Phase or Null-Phase Low-Pass Filter Well Suited for a Post Decimation Step

In this section, after introducing the MSE from the signal processing point of view, the alternative to the CG procedure is presented.

### 2.1. Multiscale Entropy: A Measure of Complexity

Twenty years ago, the MSE was proposed by Costa et al. [7] to evaluate the complexity of a signal. It consists of summing the SE of the signal itself but also the SEs of the time series derived from a CG procedure applied on the signal at different scales. The $\left\lfloor \frac{N}{\tau }\right\rfloor$ samples $y_{n,k}^{\left(\tau \right)}$ are deduced by computing the following arithmetic mean of the samples of the signal *x*:

(4) $$y_{n,k}^{\left(\tau \right)}=\frac{1}{\tau }\sum_{i=(n-1)\tau +k}^{n\tau +k-1}x_{i}\;\;\mathrm{for}\;k=1,\ldots ,\tau$$

It should be noted that $y_{n,1}^{\left(1\right)}=x_{n}$.

Applying the CG on the signal *x* with a factor τ amounts to constructing the vector ${\underline{y}}_{k}^{\left(\tau \right)}$ and stacking the values $\left\{y_{n,k}^{\left(\tau \right)}\right\}$ with $n\in \{1,\ldots ,\left\lfloor \frac{N}{\tau }\right\rfloor \}$.

From a signal processing point of view, the following two steps define the CG:

**Step 1: Applying on the signal *x* an averaging filter** defined by a causal FIR $h_{n}^{\left(\tau \right)}=\frac{1}{\tau }$ for n=0,…,τ−1 (with $\tau \leq \tau_{max}$ being the maximum scale) and zero elsewhere. The resulting transfer function H(z), which is the z transform of $h_{n}^{\left(\tau \right)}$, satisfies
  (5) $$H^{\left(\tau \right)}\left(z\right)=\left\{\begin{array}{c} \frac{1}{\tau }\sum_{n=0}^{\tau -1}z^{-n}=\frac{1}{\tau }\frac{1-z^{-\tau }}{1-z^{-1}}\;\mathrm{if}\;z\neq 1\\ 1\;\mathrm{if}\;z=1 \end{array}\right.$$
  Consequently, the frequency response of this causal filter for the normalized frequency $f\in [-\frac{1}{2},\frac{1}{2}[$ is given by
  (6) $$|H^{\left(\tau \right)}\left(f\right)|=\left\{\begin{array}{c} \frac{1}{\tau }|\frac{\sin(\pi f\tau )}{\sin(\pi f)}|\;\mathrm{if}\;f\neq 0\\ 1\;\mathrm{if}\;f=0 \end{array}\right.$$
  It is a low-pass filter. The zeros of $H^{\left(\tau \right)}\left(z\right)$ are equal to $z=e^{j2\pi \frac{k}{\tau }}$, with k∈{0,⋯,τ−1}, and are located on the unit circle in the z plane. This means that the frequency response of the filter totally rejects the normalized frequencies $\frac{k}{\tau }$ for k∈{0,⋯,τ−1}. Finally, due to the symmetry of the impulse filter, the filtering has the advantage of having a linear phase, thereby leading to a constant group delay and no phase distortion of the signal in the pass band. Only the steady state is considered. In other words, the first τ−1 samples of the output filter are not considered.
**Step 2: Decimating by a factor τ the filter output:** The samples whose indices are multiples of τ are kept for $n\in \{0,...,\left\lfloor \frac{N}{\tau }\right\rfloor -1\}$ and k∈{0,⋯,τ−1}:
  (7) $$y_{n,k}^{\left(\tau \right)}=y_{n\tau +k}$$
  In the following, ${\underline{y}}_{k}^{\left(\tau \right)}$ is the vector storing the values $y_{n,k}^{\left(\tau \right)}$, with $n\in \{0,...,\left\lfloor \frac{N}{\tau }\right\rfloor -1\}$. It should be noted that in the frequency domain, due to the decimation, the normalized frequencies above $\frac{1}{2\tau }$ will be a source of aliasing. As the frequency responses of the filters defined above have their main lobes between $-\frac{1}{\tau }$ and $\frac{1}{\tau }$ and the side lobes are not necessarily much attenuated (See (6)), the filters are not well suited to be antialiasing filters. Hence, this can be a source of problems, as reported by Valencia [25], Humeau [24], or more recently Zhao [26].
**Step 3: Computing the SE for each scale and summing them**: the SE is computed for each scale, and the MSE is defined by the sum $\sum_{\tau =1}^{\tau_{max}}SE\left({\underline{y}}_{1}^{\left(\tau \right)},1,m,r\right)$. This can be rewritten as
  (8) $$\begin{array}{c} MSE\left({\underline{x}}_{1},\tau_{max},m,r\right)=-\sum_{\tau =1}^{\tau_{max}}\log\frac{P_{r}^{m+1}\left({\underline{y}}_{1}^{\left(\tau \right)}\right)}{P_{r}^{m}\left({\underline{y}}_{1}^{\left(\tau \right)}\right)} \end{array}$$
  Note that only one decimated sequence, namely ${\underline{y}}_{k}^{\left(\tau \right)}$ with k=1, is used in the standard MSE. In addition, one of the problems of the MSE algorithm is the use of the same tolerance level *r* for all scales [29].

### 2.2. Our Theoretical Contribution

We propose to derive an MSE by taking care of the filtering step. The first step of the MSE described above is hence replaced by the following step, while the others remain unchanged:

**New step 1: Applying on the signal *x* a linear-phase or null-phase low pass filter.** For each scale $\tau \in [2,\ldots ,\tau_{max}]$, the goal is to select a low-pass causal FIR filter with a linear phase or a null phase and a normalized cutoff frequency that is smaller than $\frac{1}{2\tau }$.

This can be done by using the window method for a causal linear-phase FIR filter design or Remez algorithm.

**About the window method:** It operates with the following steps: defining the specifications of the low-pass digital filter H(f), taking the inverse Fourier transform, and windowing the resulting impulse response to obtain the FIR of the low-pass filter.
  **About the Remez algorithm:** Also known as the Parks–McClellan optimal equiripple FIR filter design, the Remez algorithm is a curve fitting method proposed in the 1970s minimizing the error between the actual frequency response and the frequency response of the designed filter. The starting point of the approach is that the frequency response of a filter whose impulse response is symmetric and whose order is odd can be expressed as a linear combination of cos(2πnf) or equivalently as a linear combination of Chebyshev polynomials of the first kind in cos(2πf). Therefore, this frequency response can be modeled as a polynomial in cos(θ). The next step is to search the coefficients of the polynomial that best approximate this frequency response so that the error between both frequency responses in the pass and stop bands are minimized. Using the alternation theorem (a polynomial fit of degree *n* to a set of bounded points is said to be minimax if and only if it attains its maximal error at *n* + 2 points with alternating signs), the normalized frequencies leading to the maximal errors and the polynomial coefficients are estimated in an iterative way. Usually less than 15 iterations are required.

An alternative is to design an IIR filter using the bilinear transform and then to design a zero-phase linear filter. Two types of processing chains can be considered:

**First strategy:** The signal $x_{n}$ is filtered by a filter whose real impulse response is $h_{n}$, thus leading to the signal $g_{n}$. Then, the time-reversed version $g_{-n}$ is filtered by a filter whose impulse response is still equal to $h_{n}$. The output is denoted as $r_{n}$. Finally, $r_{n}$ is time reversed to obtain the filtered signal of interest, which is denoted as $y_{n}$. Let $H\left(f\right)=|H\left(f\right)|\exp\left(j\Phi (f)\right)=H^{*}\left(-f\right)$; one has
  (9) $$\left\{\begin{array}{c} G(f)=H(f)X(f)\\ R(f)=H(f)G(-f)\\ Y\left(f\right)=R\left(-f\right)={\left|H\left(f\right)\right|}^{2}X\left(f\right) \end{array}\right.$$
  The Fourier transform of the impulse response of the equivalent filter is given by
  (10) $$H_{equ}\left(f\right)=|H_{equ}\left(f\right)|\exp\left(j\Phi_{equ}\left(f\right)\right)={\left|H\left(f\right)\right|}^{2}$$
  It is hence a null-phase filter ($\Phi_{equ}\left(f\right)=0$) characterized by $|H_{equ}{\left(f\right)|=|H\left(f\right)|}^{2}$.
**Second strategy:** The sequences $x_{n}$ and $x_{-n}$ are filtered by the same filter whose impulse response is $h_{n}$. This leads to two sequences respectively denoted as $g_{n}$ and $r_{n}$. The final output is equal to $g_{n}+r_{-n}$. In that case, the Fourier transform of the output is $Y\left(f\right)={2|H\left(f\right)|}^{2}\cos\left(\Phi (f)\right)$. It is a zero-phase filter if $\Phi \left(f\right)\in [0,\frac{\pi }{2}[$ in the pass band.

As there is no guarantee this is always the case, only the first strategy is considered. Note that the filter can be either FIR or IIR. For IIR, and without being exhaustive, different filter families exist such as the Butterworth filters, whose order can be large due to the constraint of a maximally flat magnitude in the pass band, and the Chebyshev filters, which are known to exhibit equiripple either in the pass band (type I) or in the stop band (type II).

The specifications of the low-pass filter frequency response have to be defined. It consists of choosing the transition bandwidth $\Delta f=f_{s}-f_{p}$, where $f_{s}$ and $f_{p}$ respectively denote the normalized stop-band corner frequency and the normalized pass-band corner frequency. For the window method and Remez approach, $f_{p}$ and $f_{s}$ are defined as $\frac{1}{2\tau }-\Delta f/2$ and $\frac{1}{2\tau }+\Delta f/2$, respectively. For the null-phase filter, $f_{p}$ is defined as $\frac{1}{2\tau }+10^{-3}$ and $f_{s}$ as $f_{p}+\Delta f$. Moreover, the maximum permissible pass-band loss $R_{p}$, as well as the stop-band attenuation $R_{s}$, have to be defined.

In the Section 3, three applications of the MSE and our proposed variants are presented.

## 3. Applications

### 3.1. Application on the Sum of Sinusoids: Interpretation and Comments

This section aims to analyze how the SE evolves with the scale τ to better understand the results one obtains when applying the MSE on basic signals. As recalled in (2), $SE({\underline{x}}_{1},\tau ,m,r)$ depends on the ratio between $P_{r}^{m+1}\left(\left[\begin{array}{ccc} x_{1} & x_{1+\tau }\cdots & x_{1+\left\lfloor \frac{N-1}{\tau }\right\rfloor \;\tau } \end{array}\right]\right)$ and $P_{r}^{m}(\left[\begin{array}{ccc} x_{1} & x_{1+\tau }\cdots & x_{1+\left\lfloor \frac{N-1}{\tau }\right\rfloor \;\tau } \end{array}\right]$

**Figure 1:**
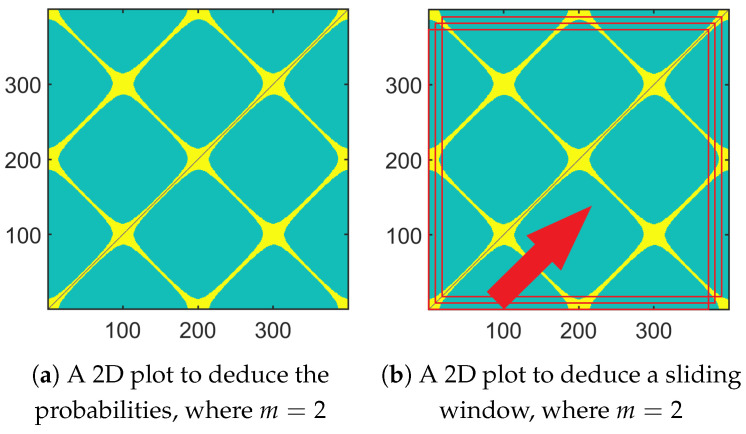
Way to compute the probability based on a geometric interpretation when using the standard SE (**a**) and when using the SE with a sliding window (**b**). The abscissa and ordinate correspond to the indices of the vectors of size *m* to be compared.

In the following and more particularly in the Figure 1a,b, Figure 7 and Figure 8, we propose to address the computation of these probabilities and consequently the SE through a geometric approach by considering a 2D representation: The pixel located at the *i*th column and the *j*th row corresponds to the Chebyshev distance between the *i*th and *j*th vectors storing *m* consecutive samples of the data. As an example, when τ=1 and m=2, the pixel located at the 2nd column and the 5th row corresponds to the Chebyshev distance between the vector storing the samples $x_{2}$ and $x_{3}$ and the vector storing the samples $x_{5}$ and $x_{6}$. When the distance is smaller than *r*, the pixel is yellow, whereas it is turquoise when it is greater. The blue pixels correspond to the case when one vector is compared with itself (hence, this is the antidiagonal in the image. This case is not taken into account when the SE is computed. This is the reason why the corresponding pixels have another color). Computing the probabilities amounts to counting the number of yellow pixels. In that case, if $d_{i,j}^{\left(m\right)}$ denotes the Chebyshev distance between the *i*th and *j*th vectors of dimension *m*, one has

(11) $$d_{i,j}^{\left(m+1\right)}=\max\left(d_{i,j}^{\left(m\right)},d_{i+1,j+1}^{\left(m\right)}\right)$$

This amounts to saying that

(12) $$d_{i,j}^{\left(m+1\right)}<r⟺d_{i,j}^{\left(m\right)}<r\;\mathrm{and}\;d_{i+1,j+1}^{\left(m\right)}<r$$

Therefore, due to (12), a pixel whose coordinates are (i,j) is yellow when the size of the vectors is equal to m+1 if and only if both pixels at coordinates (i,j) and (i+1,j+1) are yellow when the size of the vector is equal to *m*.

In the rest of this subsection, three cases are studied. Moreover, three approaches are used: the standard CG-based MSE, the variant based on a Chebyshev filter, and the variant we propose based on the null-phase filter.

**First case:** Let us first look at the case of N=400 samples of one sinusoid defined as follows:
  (13) $$x_{k}=2\cos\left(2\pi f_{1}k\right)$$
  with the normalized frequency $f_{1}=0.005$. Due to (13), the minimum and maximum values of the signal under study are obtained when *k* is a multiple of 100.

Let us now look at the corresponding 2D representation of the Chebyshev distances in Figure 1a, where m=2 and τ=1. In that case, N−m+1 vectors of size *m* have to be compared. Moreover, the abscissa and the ordinate in the 2D plot can vary between 1 and N−m+1=399. The antidiagonal necessarily corresponds to blue pixels. The pixels located along the main antidiagonal (i=j) are yellow because the sets of vectors to be compared can have samples in common. This phenomenon is retrieved along the sub-antidiagonals, whose index is a multiple of $\frac{1}{f_{1}}$ (i.e., j=±200k+i with k=0,1,…). Due to the symmetry of a sinusoid around its minima and maxima, one can also observe yellow straight lines that are orthogonal to these antidiagonals (i.e., when j=200k−i) in the 2D plot. The intersections correspond to the locations of the minima and maxima of the sinusoid. The rest of the pixels in Figure 1a are turquoise, since the Chebyshev distance is smaller than the threshold *r*.

**Figure 2:**
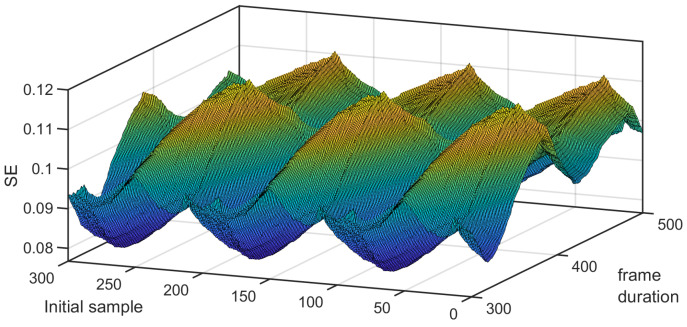
SE computed for the signal under study for different numbers of samples available and different initial values.

**Figure 3:**
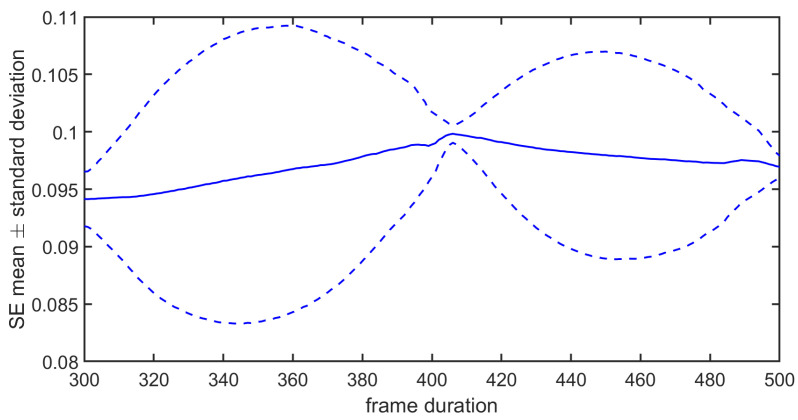
Mean ± its standard deviation of SE for different frame lengths.

*Remark on the sensitivity of the SE to the set of samples that are used:* The value of the SE was sensitive to the number of samples available and/or to the initial sample. This is due to the fact the probabilities $P_{\tau }^{m}$ and $P_{\tau }^{m+1}$ (or similarly the numbers of yellow pixels for m=2 and m=3) can change when the number of samples is modified and/or when the initial sample changes. In Figure 2, one can see that the SE varied with the number of samples available (from 300 to 500) and the first sample that was considered (from 1 to 300). We have the mean and the standard deviation of the SE computed on the frames for a given length *N* but whose initial sample changes are shown in Figure 3. One can notice that both statistics can vary considerably. Therefore, to make the computation of the SE less sensitive to the first sample, we suggest using a sliding window, computing the SE for each frame, and averaging them. An illustration is given in Figure 1b, where the window length was equal to $N_{w}=380$. As $N_{w}<N$, the resulting 2D plot is depicted by a red square. When the analysis window was shifted, the red square moved. In that case, the computational cost can be optimized. Indeed, by taking into account the computation of the probability using the 2D plot, one can just consider the upper triangular part (without the main antidiagonal). This provides the cardinal of the sample space, i.e., $\frac{\left(N_{w}-m+1\right)\left(N_{w}-m\right)}{2}$. When the analysis window was shifted by one sample, this amounted to shifting the upper triangular part corresponding to the last $N_{w}-m-1$ columns and the first $N_{w}-m-1$ rows to the left and down. Only $N_{w}-m$ new distances must be computed to deduce the event space. As the number *N* of samples available is assumed to remain the same, there may be $N-N_{w}+1$ SEs that can be computed and averaged. The resulting computation cost is equal to $\frac{N_{w}-m}{2}\left(2N-N_{w}-m+3\right)$. The number of SEs to be computed for the averaging, as well as the time shift, are chosen by the practitioner. It should be noted that this averaging is different from the cMSE. See (A3). Indeed, for the scale τ=1, the SE computed in the cMSE is the SE, whereas it is an average in this variant. In the cMSE, if τ=2 and *N* is even, there are $\frac{N}{2}$ samples in the first decimated filter output and $\frac{N}{2}-1$ samples in the second one to compute the SE and average them. In this variant, the number of samples per decimated sequence is always the same. The way to compute the probabilities for a given time shift (that is not equal to 1 but that is larger) is given in Figure 1b.

The above suggestion is not applied in the rest of this subsection in order to analyze each phenomenon separately.

**Figure 4:**
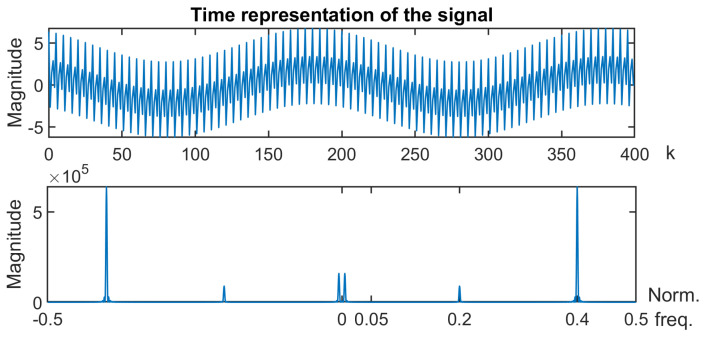
Time representation and spectrum of the signal under study.

**Second case:** Let us now address the case of N=400 samples of a sum of three sinusoids defined as follows:
  (14) $$x_{k}=2\cos\left(2\pi f_{1}k+\frac{\pi }{5}\right)+1.5\cos\left(2\pi f_{2}k+\frac{\pi }{3}\right)+4\cos\left(2\pi f_{3}k\right)$$
  with normalized frequencies $f_{2}=0.2$ and $f_{3}=0.4$. The time representation of the signal and its spectrum are given in Figure 4.

**Figure 5:**
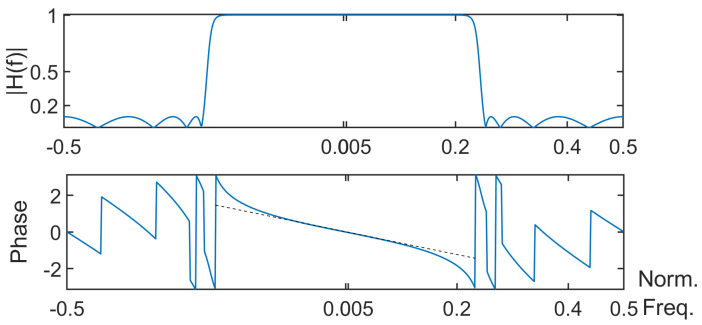
Frequency response of the Chebyshev filter: modulus and argument of H(f).

Moreover, the scale τ was set at 2, and the frequency response of the type-II Chebyshev filter is given in Figure 5. One can notice that the filter does not have a linear phase. Indeed, the phase curve should follow the black dashed line when the normalized frequency is in the interval [−0.22,0.22].

**Figure 6:**
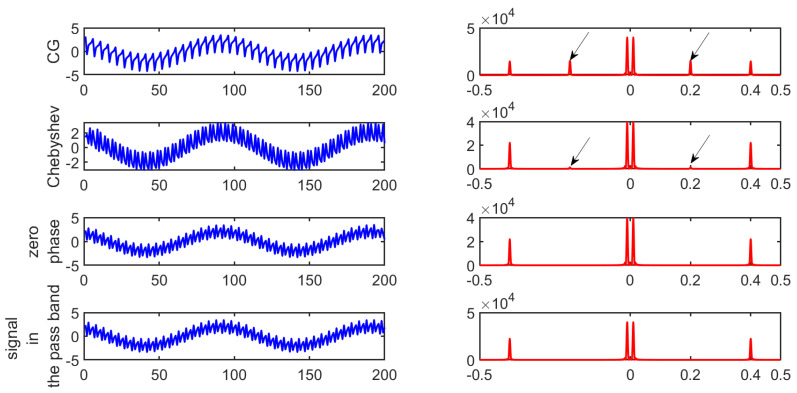
Time representation and spectrum of the signal at the scale τ equal to 2 when using CG, a Chebyshev filter, or the zero-phase filter based approach with their corresponding spectrum. The resulting signals are compared with the part of the signal located in the pass band.

When applying the CG case, aliasing occurred. When considering a low-pass filter whose cutoff frequency is well suited, this should be no longer the case in theory. However, a filtering- phase distortion occurred. Moreover, depending on the level of attenuation in the stop band, there may be a small amount of aliasing due to the decimation that follows. When using a zero-phase filter based approach, the above phenomena were greatly alleviated, as shown in Figure 6.

**Figure 7:**
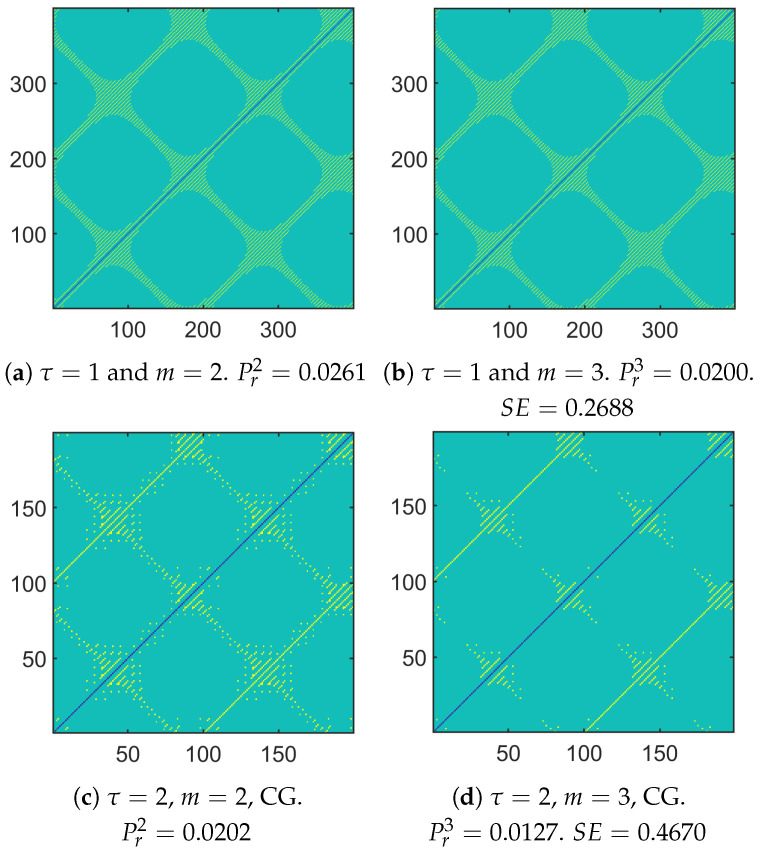
2D plot to deduce the probabilities and then computing the SE: CG-based approach.

When looking at Figure 7a with τ=1, one can notice the influence of the phase at the origin of the component whose normalized frequency is $f_{1}$. There was a global shift down and to the left when comparing Figure 7a with Figure 1a. Moreover, the two components whose normalized frequencies are $f_{2}$ and $f_{3}$ had an impact on the 2D plot by modifying the initial pattern presented in Figure 1a. Depending on $f_{2}$ and $f_{3}$ and the magnitude of both components, only segments of yellow straight lines remained in the 2D plot. The gap between them can be related to $\frac{1}{f_{2}}$ and/or $\frac{1}{f_{3}}$.

**Figure 8:**
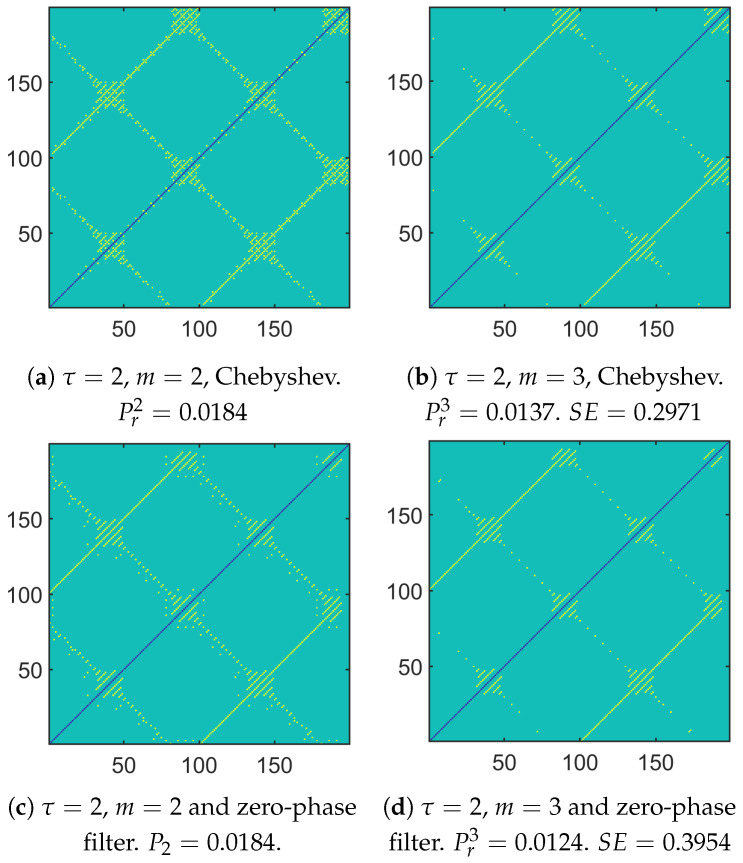
A 2D plot to deduce the probabilities and then computing the SE: Chebyshev-based approach and zero-phase filter-based approach.

Let us now compare Figure 7c,d and Figure 8, which provide the 2D plots of the vector distances when applying a CG, the Chebyshev filter, and our approach, respectively. One can see how the number of yellow pixels changed. Some yellow pixels disappeared when applying our approach, especially in the upper right corner. As a consequence, depending on the approach that is considered, there may be some differences in the probabilities and consequently in the SEs that are obtained.

### 3.2. Application on White Noises and 1/f Process

In this section, we propose to follow the same methodology as the one used in [7,25] by providing the way the entropy measure evolves with the scale for white noises and 1/f noise based on a certain number of trials.

**Remark** **1.** *Some authors study how entropies evolve with respect to the scale. Thus, the multiscale PE was applied to dfGn [30]. According to the analysis made by the authors, the PE value of a dfGn was shown to be invariant to the time scale. In [31], the authors analyzed how the $K_{2}$ entropy evolves with the scale for dfGn. They showed that it can be approximated by an affine function of the logarithm of the scale, whose slope is equal to H−1, with H being the Hurst exponent measuring the long-term memory of the time series.*

As the approaches we propose depend on the specifications of the low-pass filter, we looked at the way the curves varied with one of the specification parameters. They were compared with the curves obtained through the standard MSE [7].

**Figure 9:**
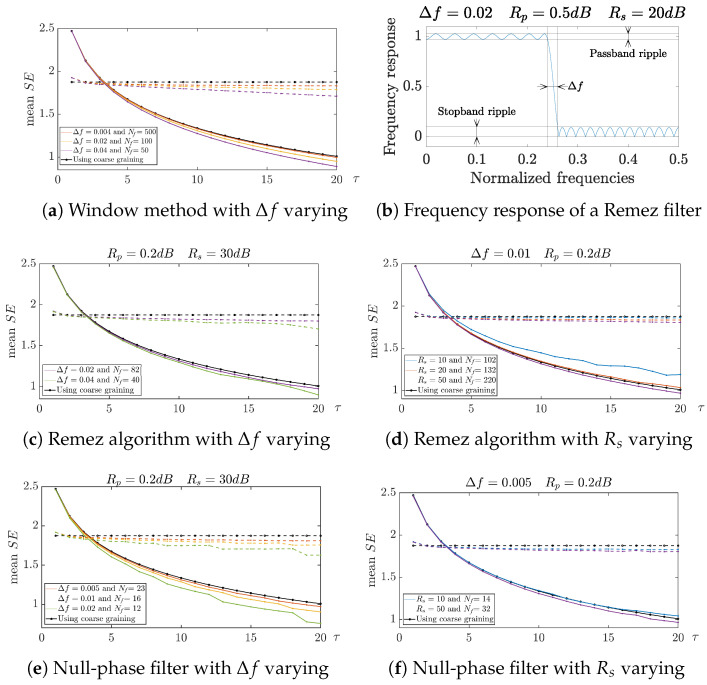
Evolution of the SE according to τ for different choices of Δf and $R_{s}$. White noise:—. Pink noise: – – [28].

Figure 9, $2N_{f}+1$ denotes the length of the impulse response for the window method (Figure 9a) and the Remez algorithm (Figure 9c,d), whereas it denotes the order of the type-II Chebyshev filter for the null-phase filter structure (Figure 9e,f). The approaches were performed on 50 signals of length 30,000. The results were then averaged to obtain the curves.

According to our simulations, when $R_{p}$ was modified in the design of the IIR filter, it did not change the shape of the curve much. For this reason, we do not present it in Figure 9. When $R_{s}$ was taken too small, the linear-phase or null-phase low-pass filter did not filter the frequencies in the stop band enough. Therefore, like the standard MSE, spectrum overlapping may occur. The resulting evolution of the entropy measure tended to the one obtained with the standard MSE. When the transition bandwidth became too wide, the stop band became smaller. Some frequencies in the transition band were not attenuated enough. Therefore, the resulting curve did not differ much from the curve obtained with the standard CG-based approach.

The reader should not conclude that obtaining curves that are different from the one obtained with CG is bad. Rather, our purpose is to show how the curve is modified when using our approach.

### 3.3. Application on Real Data to Detect Attentional Tunneling

In this section, the CG-based MSE and the new variant based on antialiasing linear-phase or null-phase filtering has been applied on real data. The goal is to detect a degraded physiological and/or cognitive state of an aircraft pilot or crew, such as attentional tunneling. It is defined as “the allocation of attention to a particular channel of information, diagnostic hypothesis or task goal, for a duration that is longer than optimal, given the expected cost of neglecting events on other channels, failing to consider other hypotheses, or failing to perform other tasks” [32]. The risk associated with this state is that the pilot may neglect new crucial information.

This attentional tunneling, which is responsible for almost all fatal controlled flight into terrain (CFIT) accidents [33], can be distinguished by two main variants: inattentional blindness and inattentional deafness [34,35]. Readers can refer to [36] for an in-depth study of attentional tunneling in the aeronautical domain. Inattentional blindness can be defined as an individual’s tendency to not perceive changes in a visual scene [37]. This state can occur in situations of high cognitive demand [38] and for a subject heavily engaged in a task [39]. Various physiological measures have been proposed to characterize this state using electroencephalogram (EEG) [40] and electrocardiogram (ECG) [41] signals. In this paper, the study is about ocular signals for two main reasons:

- Ocular data have proven to be relevant to characterize tunneling [42]. It has been shown that this state is correlated with a reduction in the number of eye movements (saccades) and an increase in fixation duration.
- Ocular data are collected from cameras or eyetrackers, which are sensors that can be accepted by a user in ecological conditions, unlike electrodes for example. This facilitates the integration of a visual tunneling monitoring solution.

The rest of this section is organized as follows: The protocol to collect the data is first presented. Then, the different methods are used for classification. We will see if the new variant of the MSE is more relevant or not.

#### 3.3.1. Information about the Protocol

**Figure 10:**
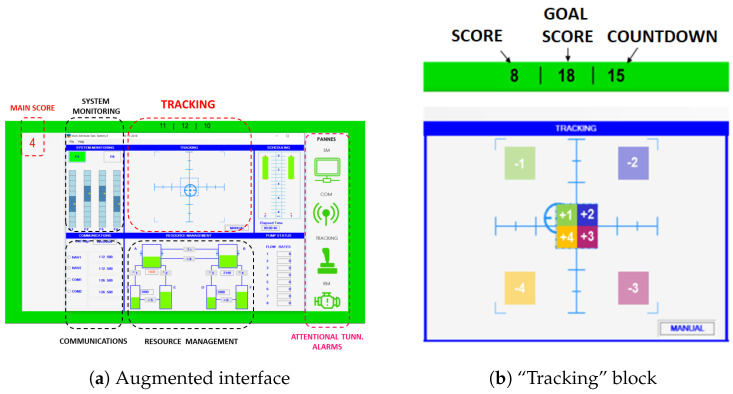
Presentation of the augmented interface used for the protocol.

Let us briefly describe the protocol. Due to its similarities with the activities of a pilot in a cockpit, the NASA MATB-II software was considered as a multitask simulator [43]. As no previous knowledge of piloting is required to use the MATB-II software, any subject with no previous piloting experience can participate in the experiment. It consists of different blocks: system monitoring, communications, resource management, and tracking. Among the various tasks that can be done, the “tracking” task is considered as the primary one, because continuous involvement is required compared with the others. To facilitate the appearance of tunneling, a scoring interface was coded and added above the “tracking” task, thus leading to an augmented interface. See Figure 10a. The user must obtain the “goal score” by placing the cursor in a specific area of the task block and clicking the joystick trigger. Four areas near the center increase the score (from one to four points), while four others (in the periphery) decrease it (from one to four points). The user must reach the goal in fifteen seconds to increment the main score (located at the top left of the interface in Figure 10b) by one point. When the user achieves it, a new goal score is proposed. Otherwise, the main score is decremented by one point and the timer is reset. See Figure 10.

**Figure 11:**
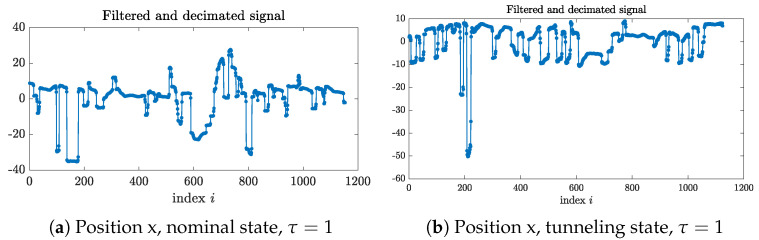
Examples of a “position x” signal.

An eye tracker (VT2 model from the company EyeTech, with a sampling rate at 40 Hz) was used to collect ocular data, namely the horizontal and vertical position of the subject gaze on the screen denoted as “position x” and the “position y”, respectively. See Figure 11. Our purpose is to study if an ocular metric, namely the SE of the gaze direction at different scales, can be used to distinguish the nominal states from the tunneling ones.

The protocol is composed of three phases.

1. During the 30-min training phase, the subject becomes familiar with each task that can be done with the simulator. Then, the subject faces a situation combining several tasks during 4 min.
2. During the reference phase, subjects are first asked to relax and do nothing during two minutes. Then, a two-minute scenario leading to a “Low” mental workload is launched.
3. During the experimental phase, there are two parts with a short break in between. Each part is composed of a task of “Low” mental workload lasting four minutes, a four-minute scenario corresponding to “Medium” mental workload, and a final task of “High” mental workload. The order of the three tasks is randomly set.

Twenty subjects filled in a NASA-TLX form [44] to subjectively evaluate their mental workload during the scenario that was proposed.

In the context of this experimentation, an occurrence of visual tunneling is detected when an alarm from the “system monitoring” task goes unnoticed, and no interaction with secondary tasks is performed for a duration exceeding twenty seconds. The data used for this analysis consist of an aggregation of performance metrics across various tasks within the NASA MatB-II software.

Among the twenty participants, five had to be removed due to technical issues. Half of the participants were observed to undergo a state of visual tunneling at least once, thuw resulting in the identification of fifteen occurrences of this state. Notably, some subjects experienced visual tunneling multiple times.

#### 3.3.2. Using CG-Based MSE or Its New Variant

**Figure 12:**
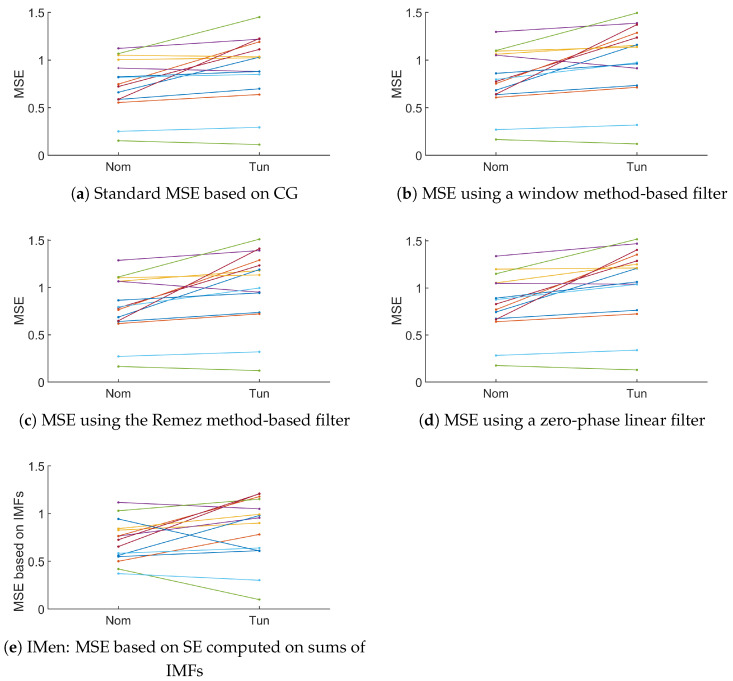
Evolution of the MSE from the nominal to the tunneling states using the standard MSE based on CG and the variants using different low-pass filter design with linear-phase or null-phase (Δf=0.02 and $R_{s}=30$ dB). Comparison with the MSE based on the IMen. τ or number of IMS equal to 3. Each color corresponds to one participant.

Based on the data collected for the fifteen subjects and during the different phases of the protocol, the next step was to use the CG-based MSE and three variants that we have proposed based either on the window method, Remez algorithm and the null-phase filter, or applied on the position x (i.e., the horizontal gaze position). In every case, instead of considering the standard MSE, the averaging used in the cMSE was considered. The purpose is to see whether the two classes, namely the nominal states and the tunneling ones, could be distinguished. According to Figure 12, most of the MSEs increased from the nominal state to the tunneling one (four decreased when using the standard CG, whereas two to three decreased when using the variants we proposed). Depending on the variant of the MSE, the values of the marker varied. In addition, the intrinsic mode entropy (IMen), i.e., the MSE based on the decomposition of the signal using EMD, is provided in Figure 12e. However, even when the IMEn increased for some participants, it also decreased for four of them quite significantly. At first sight, it is less easy to identify a trend when IMen was used.

**Figure 13:**
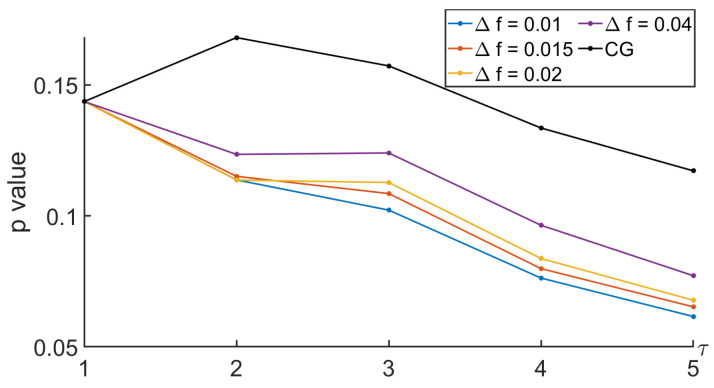
*p* value when comparing MSEs between nominal and tunneling states with different values of Δf, with the window method filter, and with $R_{s}=30$ dB.

**Figure 14:**
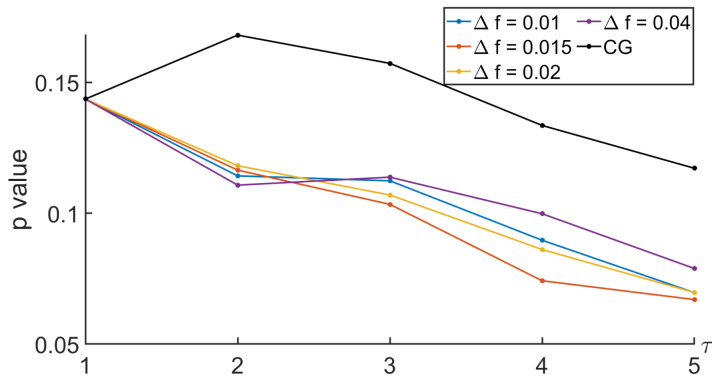
*p* value when comparing MSEs between nominal and tunneling states with different values of $\Delta_{f}$, with Remez filter, and with $R_{s}=30$ dB.

**Figure 15:**
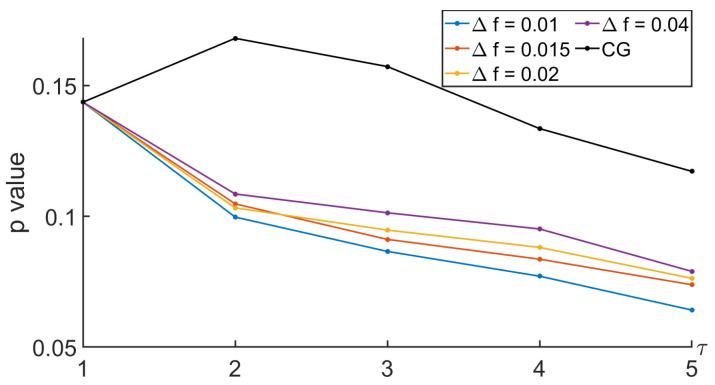
*p* value when comparing MSEs between nominal and tunneling states with different values of Δf, with a null-phase filter, and with $R_{s}=30$ dB.

**Figure 16:**
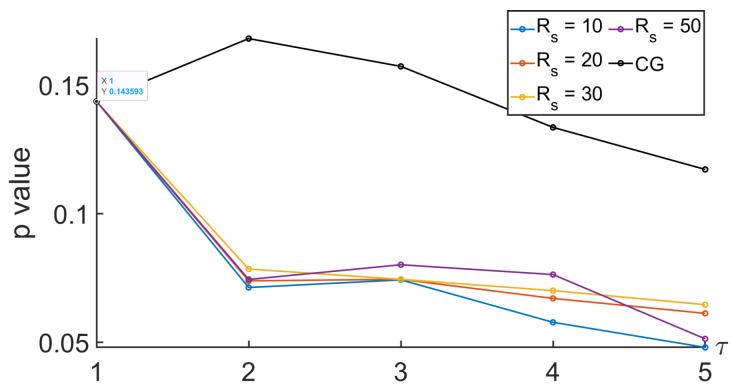
*p* value when comparing MSEs between nominal and tunneling states with different values of $R_{s}$, with a null-phase filter, and with Δf=0.005.

To analyze the relevance of the approaches, the comparison between the MSE computed during the nominal and the tunneling states was based on the Behrens–Fisher problem. It is a hypothesis testing dealing with the difference between the means of two normally distributed populations when the variances of the two populations are not assumed to be equal based on two independent samples. The indicator we are interested in is the *p* value. It is a score between 0 and 1 indicating whether or not two sets come from distributions having the same mean. Thus, a low *p* value indicates that there is low probability that the two sets come from equal mean distributions. Thus, a low *p* value indicates that the two sets can easily be differentiated. Regarding our approaches based on the null-phase filter, the Remez approach, or the window method, we proposed to see how the *p* value evolved with respect to some specifications of the filter such as Δf and $R_{s}$. See Figure 13, Figure 14, Figure 15 and Figure 16.

In Figure 13, one can see that the *p* values decreased with the scale τ. Using the MSE up to a scale τ equal to 5 should be better than using the MSE up to a smaller scale. In addition, when studying the influence of the specification parameters used to design the linear-phase low-pass filter with the window method, the smaller Δf was, the more distinct the pass band and the rejected band were and the smaller the *p* value was. Finally, the *p* values were much smaller than those obtained on this test when comparing the MSEs computed with the CG-based MSE.

In Figure 14, similar comments can be made when analyzing the way the *p* value evolved with the scale and Δf, which is one of the parameters defining the low-pass filter designed with the Remez algorithm.

In Figure 15 and Figure 16, we propose to look at the sensitivity of the *p* value when the parameters of the filter used to get the null-phase low-pass filter were modified. Firstly, $R_{s}$ was set at 30 dB, and Δf varied. Then, Δf was equal to 0.005, and different values of $R_{s}$ were considered. Once again, the *p* value decreased with the scale for τ≤2. The smaller the Δf, the smaller the *p* value. Therefore, our variant was more reliable than the MSE based on a CG.

## 4. Conclusions and Perspectives

To replace the standard coarse-graining step of the MSE that induces aliasing, a linear-phase FIR filter and a null-phase structure based on an IIR filter have been used in our first variant. Following the same type of analysis as in [7,25], the sample entropies of white noise and 1/f noise at different scales were studied. As the parameter of the filters could be tuned by the practitioner, our simulations confirmed that if the stop-band ripple is too small or if the transition bandwidth of the filter is too wide, the antialiasing properties of the filter become lesser. As the FIR filter order may be large, it may be less relevant when the number of samples available is low, and we suggest using the null-phase filter structure. Our proposal, based on a linear- or null-phase filter, seems moderate from the signal processing point of view, but the resulting MSE could serve a large community of users. It shows its relevance in the application dedicated to attentional tunneling.

## Figures and Tables

**Figure A1:**
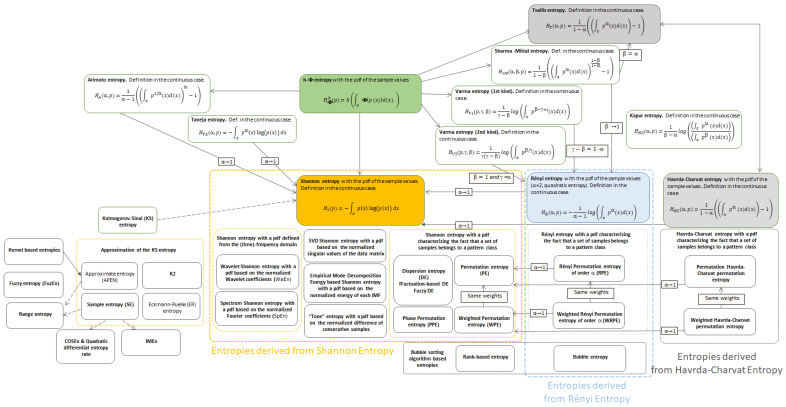
Links between the various entropies.

**Figure A2:**
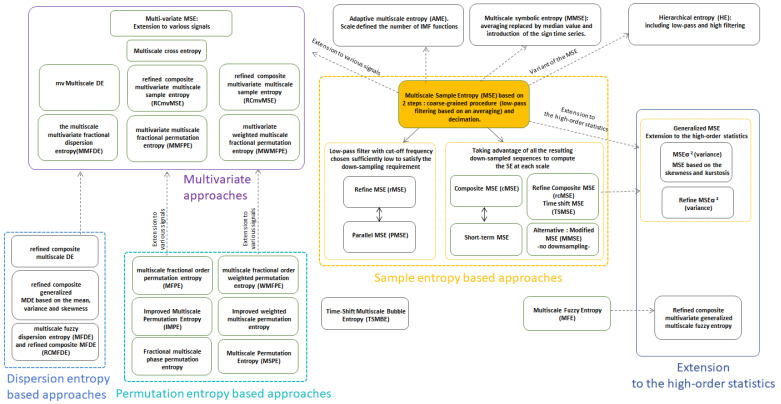
Multiscale entropies and its variants.

## Data Availability

Data are contained within the article.

## Abbreviations

The following abbreviations are used in this manuscript:

AME	adaptive MSE
ApEN	approximate entropy
AR	autoregressive
ARFIMA	autoregressive fractionnaly integrated moving average
ARMA	autoregressive with moving average
CFIT	controlled flight into terrain
CG	coarse-graining
CMSE	composite MSE
COSEn	coefficient of the sample entropy
dfBm	discrete fractional Brownian motion
dfGn	discrete fractional Gaussian noise
DE	dispersion entropy
EMD	empirical mode decomposition
ECG	electrocardiogram
EEG	electroencephalogram
ER	Eckmann–Ruelle
FI	fractionally integrated
FIR	finite impulse response
FuzEn	fuzzy entropy
HE	hierarchical Entropy
IIR	infinite impulse response
IMEn	intrinsic mode entropy
IMFs	intrinsic mode functions
IMPE	improved multiscale PE
KS	Kolmogorov–Sinai
LZ	Lempel–Ziv
MEMD	multivariate EMD
MFE	multiscale fuzzy sample entropy
MFPE	multiscale fractional-order PE
MGMSE	multivariate generalized MSE
MGrcMSE	multivariate generalized rcMSE
MMSE	modified MSE
MrcMSE	multivariate
MSampEn	multivariate sample entropy
MSE	multiscale entropy
MSSE	multiscale symbolic entropy analysis
mv	multivariate
mvMPE	mv multiscale PE
PE	permutation entropy
PSD	power spectral density
rcMFE	refined composite multiscale fuzzy entropy
rcMPE	refined composite multiscale permutation entropy
rcMSE	refined composite MSE
rMSE	refined MSE
SE	sample entropy
SV	singular value
SVD	singular value decomposition
TSMBE	time shift multiscale bubble entropy
WPE	weighted permutation entropy
WMFPE	weighted multiscale fractional-order PE

## Appendix A

### Appendix A.1. About Entropies

Entropy has been used in a wide range of applications, from thermodynamic to information theory via statistical mechanics and the structure of graphs. This is a measure of different properties such as the amount of disorder, uncertainty, lack of predictability, and a measure of information.

Shannon entropy has been one of the most popular entropies for more than 70 years. Then, a great deal of interest has been paid to the definitions of alternative entropies, especially in the 1960s and the 1970s with many contributors such as Arimoto and Picard when dealing with discrete random variables. Some generalizations of Shannon entropy were proposed such as Sharma–Mittal entropy where the practitioner can tune two real parameters: α≠1, which is called the order, and β≠1, which is known as the degree. When β=α, Tsallis entropy is obtained. When α tends to 0 (resp. infinity), Hartley entropy (resp. min entropy) is deduced. When α=2, one obtains the collision entropy. In addition, the Kapur entropy can be defined, up to a multiplicative factor, as the difference between the Rényi entropies for two different orders. A generalization of the entropy is given through the (h,Φ) entropy, whose simplified definition is given by using the functions *h* and Φ, which are increasing and concave, respectively (or decreasing and convex):

(A1) $$H_{\Phi }^{h}=h\left[\int \Psi \left(p\left(x\right)\right)dx\right]$$

If h(t)=t and Ψ(t)=−tlog(t), one obtains Shannon entropy. Other functions can be defined to obtain Taneja entropy, Arimoto entropy, Varma entropy of the first and second kind, Havrda, and Charvat entropy. The reader may refer to [45] for more details.

The notion of metric entropy of a dynamical system with *F* degrees of freedom, whose state measured at time instant multiples of δ that can be in the phase space partitioned into a collection of boxes of size *r*, was introduced at the end of the 1950s. This led to the Kolmogorov–Sinai (KS) entropy, whose value makes it possible to distinguish an ordered system from a chaotic one. This was the starting point of other research activities whose purpose was to obtain an approximation of the KS entropy in practice. This led to quantities such as $K_{2}$ entropy proposed by Grassberger and Procaccia, the Eckmann–Ruelle (ER) entropy, and finally the approximate entropy (ApEn) proposed by Pincus in 1991 [6]. In that case, given a set of samples ${\left\{x_{n}\right\}}_{n=1,\ldots N}$, one first computes the probability that the distance between one reference vector storing *m* consecutive values of the sample set starting by $x_{i}$ and another vector of length *m* starting by $x_{j}$ is smaller than a threshold, which is also called tolerance level *r*. Note that the vectors can start by the same sample and, hence, can be the same. For a given value of *m*, the logarithms of the N−m+1 probabilities are summed. The same procedure is done for m+1. At that stage, the ApEn is the difference between both quantities when *N* tends to infinity. It is hence defined for a given length *m* and tolerance level *r*. The ApEn has been generalized through the kernel-based entropies. See for instance [46]. The SE is another extension of the ApEn [47]. As it depends on the tolerance level *r*, the quadratic differential entropy rate can be considered where log(2r) is added. To address the problem of atrial fibrillation, one can also use the coefficient of the SE (COSEn). The latter corresponds to the SE from which one subtracts the sum of two terms: the log(2r) and the logarithm of the RR interval mean [48].

In the intrinsic mode entropy (IMEn), the authors propose to use the empirical mode decomposition (EMD) in order to extract the intrinsic mode functions (IMFs) of the signal. Then, the SEs of the cumulative sums of the IMFs are computed [49].

As an alternative to the SE, other approaches can be used. Thus, some authors like Chen et al. [50,51] suggested using the concept of Zadeh’s fuzzy sets in the classification procedure in the ApEn and the SE. This led to the fuzzy entropy (FuzEn). However, its computational cost of the FE is larger than that of the SE. In addition, when short time series are studied, this may lead to poor results.

In the range entropy [52], instead of considering the Chebyshev distance, the authors suggested using the difference between the maximum and the minimum divided by the sum of the maximum and the minimum. Note that this is no longer a distance.

Another type of approaches is based on a bubble sorting algorithm that consists of repeatedly swapping two adjacent values when they are in the wrong order to obtain values ordered in the ascending order. The rank-based entropy was also proposed in [53].

In [54], the data vectors of length *m* were sorted in an ascending order by using the bubble sorting algorithm. In each case, the number of swaps were saved to obtain an histogram. It was used to compute the second-order Rényi entropy. The standard bubble entropy (bEn) is given as the difference between the entropies computed for the vector of length m+1 and *m*. It is finally normalized by $\log(\frac{m+1}{m-1})$.

As an alternative to these types of entropies, starting from the definition of Shannon entropy, some authors have proposed to define a distribution that is different from the pdf of the data. Thus, singular value decomposition (SVD)-based entropy has been presented in [55]: in that case, the SVD of the Hankel data matrix can be computed; each singular value (SV) is then divided by the sum of the SVs, and the Shannon entropy of the resulting distribution is computed. When dealing with the EMD energy entropy, the pdf is built from the the normalized energy of each IMF [56]. In [57], the signal was first filtered using a high-pass difference filter. Then, the normalized difference between two consecutive samples was computed and expressed in percentage. The probability that the resulting index belongs to the interval [i,i+1[ with *i* an integer was computed. In [58], Fourier entropy was used, where the principle is to decompose the signal of interest in a Fourier basis. The modules of the Fourier coefficients are normalized so that the sum is equal to 1. Shannon entropy of the resulting distribution is computed. Note that a time evolving entropy based on a sliding window can be also considered. As an alternative, a wavelet-based entropy can be defined. See [59].

Regarding permutation entropy (PE) [60] based on the order relations among values of a signal, the principle is the following: when considering *m* consecutive samples whose values are assumed to be all different, m! patterns can be obtained. This number corresponds to the number of permutations that can be done on a set whose cardinal is equal to *m*. The probability that each pattern appears can then be calculated on the signal and its delayed version. This consists of counting the number of times the pattern appears and then normalizing it. Finally, Shannon entropy is evaluated. However, it is is sensitive to noise. In addition, this approach reflects how adjacent values are organized, but the data magnitudes are omitted. For this reason, the phase PE was developed [61]. It is based on the analytical signal deduced from the signal under study. In addition, the weighted PE (WPE) has been proposed. In that case, the estimation of the probability no longer consists of only counting the number of times each pattern appears. When a pattern is identified, the variance of the *m* data samples is considered instead. The variances are summed and then normalized. These approaches have been also extended to the Rényi entropy of order α [62] and to Havrda–Charvat entropy [63]. The dispersion entropy (DE) is based on a linear or a nonlinear mapping technique, the definition of the possible dispersion patterns, the probability they appear, and the computation of the corresponding Shannon’s entropy [64]. Note that the computational cost of DE is much smaller than that of SE. However, DE is sensitive to the number of classes. Some variants were recently developed: one called fluctuation-based DispEn (FDispEn) considers the differences between adjacent elements of the dispersion patterns [65], whereas the fuzzy DE combines DE and fuzzy membership functions for signal quantization and was proposed in [66].

Finally, in neuroscience and biomedical applications for instance, various signals can be recorded at the same time (electroencephalogram (EEG) for instance). For this reason, multivariate (mv) entropy can be considered. The reader may refer to [67].

### Appendix A.2. Some Variants of the MSE

In 2009, the refined MSE (rMSE) was proposed by Valencia et al. [25], where the FIR filter was replaced by an IIR low-pass Butterworth filter whose cutoff frequency is chosen to downsample the signal. Note that in 2021, the authors in [26] suggested using the same kind of filter (the resulting method was called parallel MSE by the authors). However, this filter does not have a linear phase. Therefore, the design of this filtering step must be improved.

In 2013, the composite MSE (cMSE) consisted of using the τ sequences that can be defined after the decimation step [22]:

(A2) $$\begin{array}{cc} cMSE({\underline{x}}_{1},\tau_{max},m,r) & =\sum_{\tau =1}^{\tau_{max}}cSE\left({\underline{x}}_{1},\tau ,m,r\right) \end{array}$$

where $cSE\left({\underline{x}}_{1},\tau ,m,r\right)=\frac{1}{\tau }\sum_{k=1}^{\tau }SE\left({\underline{y}}_{k}^{\left(\tau \right)},1,m,r\right)$ corresponds to the average of the sample entropies computed on the τ sequences obtained from the filtering and the decimation. Averaging has the advantage of reducing the variance of the estimated SE values. The above equation can be rewritten as follows:

(A3) $$\begin{array}{cc} cMSE({\underline{x}}_{1},\tau_{max},m,r) & =-\sum_{\tau =1}^{\tau_{max}}\frac{1}{\tau }\sum_{k=1}^{\tau }\log\frac{P_{r}^{m+1}\left({\underline{y}}_{k}^{\left(\tau \right)}\right)}{P_{r}^{m}\left({\underline{y}}_{k}^{\left(\tau \right)}\right)}\\ & =-\sum_{\tau =1}^{\tau_{max}}\log\frac{{\left(\prod_{k=1}^{\tau }P_{r}^{m+1}\left({\underline{y}}_{k}^{\left(\tau \right)}\right)\right)}^{\frac{1}{\tau }}}{{\left(\prod_{k=1}^{\tau }P_{r}^{m}\left({\underline{y}}_{k}^{\left(\tau \right)}\right)\right)}^{\frac{1}{\tau }}} \end{array}$$

What is called “short-term MSE” and proposed by [68] is described by the same process.

As pointed out in (A3), the cMSE amounts to computing the mean of logarithms or equivalently computing the log of geometric means. In contrast, the refined composite MSE (rcMSE) consists of computing the log of the arithmetic means [69]:

(A4) $$rcMSE\left({\underline{x}}_{1},\tau_{max},m,r\right)=-\sum_{\tau =1}^{\tau_{max}}\log\frac{\sum_{k=1}^{\tau }P_{r}^{m+1}\left({\underline{y}}_{k}^{\left(\tau \right)}\right)}{\sum_{k=1}^{\tau }P_{r}^{m}\left({\underline{y}}_{k}^{\left(\tau \right)}\right)}$$

The geometric mean is less sensitive than the arithmetic mean to the highest values.

In the modified MSE (MMSE) [70], the authors proposed to apply the averaging filter. Instead of decimating the filter output *y*, the sample entropies were directly computed on *y*. According to the authors, this has the advantage of increasing the number of vector pairs, but the computational cost becomes larger.

(A5) $$\begin{array}{cc} MMSE({\underline{x}}_{1},\tau_{max},m,r) & =\sum_{\tau =1}^{\tau_{max}}SE\left({\underline{y}}_{1},\tau ,m,,r\right) \end{array}$$

In [21], Pham et al., suggested introducing different time shifts on various intervals of time series. In some applications, the MSE was applied on data that had been preprocessed. See for instance [71].

The concept of multiscale can be extended to other entropy metrics such as those recalled in Section 2.1. Among them, the multiscale PE is detailed in [72]. Some variants also exist such as the refined composite multiscale PE (rcMPE) [73], the improved multiscale PE (IMPE) [74], the multiscale fractional-order PE (MFPE), and its weighted version—whose acronym is WMFPE in [75] or the fractional multiscale phase PE [76]. When fractional approaches are considered, the α derivative of the information element −log(p) with −1≤α≤1 and *p* probability is considered in the definition of the entropy instead of −log(p).

Multiscale fuzzy SE (MFE) has been derived, as well as composite and refined composite multiscale fuzzy entropy (rcMFE) [77].

In 2015, the generalized MSE was proposed by Costa et al. [78]. Instead of computing the mean in the first step of the MSE approach, a higher-order moment (for instance the variance) was considered. When dealing with the variance, one obtains the MSE$\sigma^{2}$. As the length of the CG series decreases when the scale increases, the estimation of the SE may not be accurate. For this reason, the refined MSE$\sigma^{2}$ was proposed in [79], thereby following the same methodology as in the rcMSE. The MSE based on skewness and the kurtosis have been also developed in [80].

The solution proposed by Hu et al. [81] is to decompose the signal into IMFs by using the EMD and its variant known as multivariate EMD (MEMD), which has the advantage of fixing some problems such as the the mode misalignment. As the EMD and MEMD essentially act as dyadic filter banks, the authors suggest summing a certain number of IMFs in order to obtain different sets of data, whose spectrums tend to exhibit lower and lower frequencies. The number of IMFs that are considered corresponds to a scale. Then, the SE is computed on each signal. This leads to the adaptive MSE (AME). However, this method has some limitations such as the sensitivity to the noise.

Finally, in [82], the authors suggested applying the CG procedure, where the sample average is replaced by the median value. From a signal processing point of view, the low-pass linear filter is replaced by a nonlinear filter. Then, the authors suggested deducing the sign time series. When the difference between two consecutive samples is positive, the value is equal to 1. Otherwise, it is equal to 0. As a consequence, the number of possible patterns was found to be finite, and the authors used both the SE and this second entropy. This method is called multiscale symbolic entropy analysis (MSSE).

In hierarchical entropy (HE) [8], the signal is decomposed into two sequences: the low-pass filtered one obtained by the averaging filter and the high-pass filtered one obtained by the difference filter. Then, each resulting sequence is decomposed into two sequences: a low-pass and a high-pass one. This leads to a “hiearchical” decomposition of the signal. Then, the SE is computed for each component.

Extension of other entropies to the multiscale has been done. Thus, in [83], the bubble entropy led to time shift multiscale bubble entropy (TSMBE).

In [84], the authors suggested using the refined composite multiscale DE and applying it to biomedical signals. More recently, they considered the refined composite generalized MDE based on the mean, variance, and skewness [85], and they proposed the multiscale fuzzy dispersion entropy (MFDE) and refined composite MFDE (RCMFDE) [86].

Instead of processing the signals independently, mv MSE, as well as multivariate rcMSE (MrcMSE), have been proposed [67]. They are based on multivariate SE (MSampEn). Humeau proposed to combine the above concepts to obtain what is called multivariate generalized MSE (MGMSE) and multivariate generalized rcMSE (MGrcMSE) [87]. Weighted versions were recently studied in [88]. The mv multiscale PE (mvMPE) has been also developed, as well as the mv multiscale DE [89] and the multiscale multivariate fractional dispersion entropy (MMFDE) [90]. The reader could finally notice that multiscale approaches have been also derived for crossentropy to analyze the dynamical characteristics of the coupling behavior between two sequences on multiple scales [91].

The reader can refer to Figure A1 and Figure A2 to see the links between the entropies and the variants of the MSE.
